# Redefining Cancer Screening Coverage—Screening to Diagnosis

**DOI:** 10.1001/jamahealthforum.2024.2814

**Published:** 2024-09-06

**Authors:** Crystal D. Taylor, A. Mark Fendrick, Lesly A. Dossett

**Affiliations:** Department of Surgery, University of Michigan, Ann Arbor (Taylor, Dossett); Department of Internal Medicine, University of Michigan, Ann Arbor (Fendrick).

Among the leading causes of death in the US, cancer ranks second only to cardiovascular disease. The best cancer outcomes occur when cancers are detected by screening, in which premalignant or early-stage disease is detected in asymptomatic, average-risk individuals. Screening can improve outcomes by preventing cancer (eg, removing polyps in the colon that may become malignant neoplasms) and detecting cancers at an earlier stage for which less treatment is needed.

In recognition of the need to implement policies aimed to enhance access and improve equity of cancer screening, the preventive services provision of the Affordable Care Act (ACA) requires that most health insurers provide preventive services receiving an A or B rating from the US Preventive Services Task Force (USPSTF) to individuals with no out-of-pocket costs (OOPCs). This policy expanded access and reduced disparities to many preventive services including the initial screening test for the 4 cancers that are covered (ie, breast, cervical, colorectal, and lung cancer) with zero cost sharing under the ACA provision. However, in most cases, additional diagnostic steps are necessary after an initial abnormal screening test result to make a definitive cancer diagnosis (or not). While coverage without cost sharing for the initial screening test is a positive step, some patients who learn that their first test result is abnormal—those who benefit most from screening—face substantial financial barriers to complete the required steps for a definitive diagnosis. Thus, the imposition of cost sharing upon the beneficiary can lead to delays and worse disparities in diagnosis, treatment, and patient outcomes.

## Lost in Diagnostics: The Case of Breast Cancer

Breast cancer is one type of cancer for which subsequent diagnostic testing is necessary following a screening abnormality. Patients with an abnormal 2-view screening mammogram result are recommended to undergo subsequent diagnostic imaging with further mammographic, ultrasonographic, and occasionally magnetic resonance images obtained by a diagnostic breast radiologist. Tissue sampling via image-guided or surgical biopsy may also be required if a suspicious abnormality is confirmed on diagnostic imaging.

Several factors contribute to a lack of diagnostic follow-up after an initial abnormal screening examination result, and there is a robust and growing evidence base demonstrating that cost sharing plays a role. A 2021 study reported that commercially insured women aged 40 to 64 years with job-related health insurance who received additional imaging and biopsies after an abnormal screening mammogram result between 2010 and 2017 faced nontrivial and rising OOPCs.^1^ A 2023 study demonstrated that insured patients required to pay higher OOPCs had lower utilization rates of follow-up diagnostic testing.^2^ Furthermore, a 2018 study found that women experienced delays in time to diagnostic imaging, breast biopsy, incidence of early-stage breast cancer diagnosis, and chemotherapy initiation when placed on a high-deductible health plan compared to women who remained on a low-deductible health plan.^3^ Although this study was an evaluation of patients limited to high-deductible health plans and did not follow up patients over time to evaluate differences in overall breast cancer survival, it can serve as a model for evaluating the elimination of cost sharing on cancer outcomes by assessing time to diagnosis, treatment, and short- and long-term overall and breast cancer–specific survival in groups of patients with no, low, or high OOPCs as currently no such studies exist.

In April 2024, the USPSTF released updated breast cancer screening recommendations that lowered the age at which to begin screening from 50 to 40 years, extending eligibility to no-cost screening for upwards of 20 million individuals in the US.^4^ In the updated recommendations, the USPSTF acknowledges that screening alone is insufficient: “To achieve the benefit of screening and mitigate disparities in breast cancer mortality by race and ethnicity, it is important that all persons with abnormal screening mammography results receive equitable and appropriate follow-up evaluation and additional testing, inclusive of indicated biopsies…”^4^ As screening mammograms are noninvasive tests that cannot alone diagnose breast cancer and subsequent diagnostic workup is required, efforts to eliminate cost sharing for diagnosis should be prioritized.

State-specific legislation eliminating cost sharing for diagnostic testing in breast cancer has been passed in at least 12 states, including Georgia and Maryland. Additionally, a report published by the Maryland Health Care Commission on the financial impact of eliminating cost sharing for image-guided biopsies for breast cancer using a financial model concluded that their policy change will result in over 12 000 additional mammograms and over 800 additional breast biopsies being performed, with a likely 1 to 2 in 1000 screening mammograms resulting in a diagnosis of breast cancer (some of which will be early-stage leading to more cost-effective treatment) totaling an increased cost of $0.33 per member per month and a $0.39 premium increase.^5^ At the federal level, the Access to Breast Cancer Diagnosis Act of 2023 was introduced into the US Senate in July 2023, which, if signed into law, would require group health plans and health insurers offering group or individual health insurance to cover diagnostic and supplemental breast examinations—as defined by the National Comprehensive Cancer Network guidelines, include diagnostic mammogram, magnetic resonance imaging, and breast ultrasonography—without patient cost sharing.^6^ Although this specific legislation would be a further step to enhance access and remove patients’ financial stress, the bill does not propose coverage for necessary biopsies.

## Redefining Comprehensive Cancer Screening: The Case of Colorectal Cancer

Following a multistakeholder campaign, federal policies that redefine colorectal cancer (CRC) screening and require coverage for the entire diagnostic process (eg, follow-up colonoscopy) without OOPCs have been successfully implemented in both commercial insurers (under FAQs About Affordable Care Act Implementation 51) and Medicare. These decisions were supported by research showing that a majority of commercially insured and Medicare beneficiaries who required follow-up diagnostic colonoscopy after a positive stool-based screening test result were subject to cost sharing,^7^ and a modeling study reporting that waiving OOPC for follow-up colonoscopy, assuming a 10–point percentage increase in the rates of colonoscopy screening, follow-up and surveillance, resulted in a 13% reduction in CRC deaths with an associated cost-effectiveness than if OOPCs were waived with no change in screening rates.^8^ Similarly, cost-effectiveness was seen in waiving OOPCs for follow-up colonoscopy after an abnormal stool-based examination finding.

Successful efforts to enhance access to the entire CRC screening process have made diagnostic services more accessible for many individuals who otherwise could not afford them.^9^ This strategy should be extended to other screening-detected cancers so that individuals with positive initial screening examinations—and the most to gain from screening—can reap the full benefits of prevention and early detection. A recent statement by the American Cancer Society supports this policy and explicitly states that cancer screening is not a single test but rather a continuum of tests to reach a diagnosis and that consumer cost sharing should be eliminated for recommended follow-up diagnostic testing.^10^

## Conclusions

Cancer screening is a multistep process that often requires follow-up testing to make a definitive diagnosis. The existence of barriers—financial and otherwise—that might deter the completion of the recommended diagnostic workup following an abnormal initial screening test undermines the goals of the program. Implementation of policies that eliminate OOPCs for the entire screening and diagnostic continuum is needed to improve access and enhance equitable use of these services and achieve the goals of reduced cancer-related morbidity and mortality.
